# Anti-Tissue-Transglutaminase IgA Antibodies Presence Determination Using Electrochemical Square Wave Voltammetry and Modified Electrodes Based on Polypyrrole and Quantum Dots

**DOI:** 10.3390/bios15010042

**Published:** 2025-01-13

**Authors:** Angela Gabriela Pãun, Simona Popescu, Alisa Ioana Ungureanu, Roxana Trusca, Alina Popp, Cristina Dumitriu, George-Octavian Buica

**Affiliations:** 1 Faculty of Applied Chemistry and Materials Science, National University of Science and Technology Politehnica Bucharest, 313 Splaiul Independentei, Sector 6, 060042 Bucharest, Romania; angela.olaru@upb.ro (A.G.P.); simona.popescu@upb.ro (S.P.); alisa.ungureanu@stud.faima.upb.ro (A.I.U.); truscaroxana@yahoo.com (R.T.); 2 National Institute for Mother and Child Health “Alessandrescu-Rusescu”, 120 Lacul Tei Boulevard, Sector 2, 020395 Bucharest, Romania; stanescualina@yahoo.com

**Keywords:** silk fibroin, quantum dots, polypyrrole, electroanalysis, anti-tTG antibody

## Abstract

A novel electrochemical detection method utilizing a cost-effective hybrid-modified electrode has been established. A glassy carbon (GC) modified electrode was tested for its ability to measure electrochemical tTG antibody levels, which are essential for diagnosing and monitoring Celiac disease (CD). Tissue transglutaminase protein biomolecules are immobilized on a quantum dots-polypyrrole nanocomposite in the improved electrode. Initial, quantum dots (QDs) were obtained from Bombyx mori silk fibroin and embedded in polypyrrole film. Using carbodiimide coupling, a polyamidoamine (PAMAM) dendrimer was linked with GQDs-polypyrrole film to improve sensor sensitivity. The tissue transglutaminase (tTG) antigen was cross-linked onto PAMAM using N-(3-dimethylaminopropyl)-N′-ethylcarbodiimide hydrochloride (EDC)-N-hydroxy succinimide (NHS) chemistry to develop a nanoprobe that can detect human serum anti-tTG antibodies. The physicochemical characteristics of the synthesized nanocomposite were examined by FTIR, UV-visible, FE-SEM, EDX, and electrochemical studies. The novel electrode measures anti-tissue antibody levels in real time using human blood serum samples. The modified electrode has great repeatability and an 8.7 U/mL detection limit. Serum samples from healthy people and CD patients were compared to standard ELISA kit assays. SPSS and Excel were used for statistical analysis. The improved electrode and detection system can identify anti-tissue antibodies up to 80 U/mL.

## 1. Introduction

Gluten, a protein in certain grains, damages the small intestine in celiac disease (CD) patients. CD patients typically produce autoantibodies that tear down the intestinal mucous membrane. The main test for CD is a biopsy [1]. The discovery and use of highly specific gluten-dependent tissue autoantibodies in the immunoglobulin A (IgA) class revolutionized CD diagnosis, follow up and screening [2]. The 2020 European Society for Paediatric Gastroenterology, Hepatology, and Nutrition (ESPGHAN) and the 2023 American College of Gastroenterology (ACG) guidelines recommend diagnosing celiac disease in symptomatic children without a biopsy. tTG IgA levels more than 10 times than the upper reference limit (10 U/mL) and EMA test positive indicate celiac disease, according to current criteria [3]. These autoantibodies generated in individuals with CD selectively target tissue transglutaminase (tTG) protein [1]. Fewer than 10% of cases are now detected, meaning that symptoms often go unnoticed for more than 10 years [4]. Along with lab test techniques like enzyme-linked immunosorbent assay (ELISA), scientists are still developing fast, sensitive, affordable, and easy-to-use tools for point-of-care usage. Electrochemical sensors are popular for their sensitivity, selectivity, flexibility, simplicity and reduced sample quantities leading to cost reductions [1,5]. Advances in these sensors for clinical diagnostics, food quality control, and environmental monitoring are pursued [5]. Electrochemical transduction devices are convenient, portable, sensitive, and cost-effective for point-of-care analysis [6]. Generally, transducers must have a large surface area to accommodate bioreceptors. Additionally, electrochemical sensors need increased electron transport to the electrode. Various materials, including nanoparticles, polymers and proteins are employed to change electrode surfaces [7].

Thanks to their inherent electric characteristics, conducting polymers (CPs) have found several uses in recent decades, already being considered as materials of great importance [8]. Polypyrrole (PPy) has been suggested for various in vivo biomedical uses because of its biocompatibility, high conductivity, and environmentally favorable characteristics. It is one of the most researched conducting polymers [9]. The conductivity of PPy increases considerably when is doped with nanoparticles [10]. The presence of quantum dots modifies the PPy’s properties like electrical, optical, photocatalytic, mechanical, thermal, separation, and purification capabilities [10]. Nowadays, many researchers are interested in carbonizing small molecules to create photoluminescent dots. Some examples of these nanostructures are carbon nanodots (CNDs), graphene quantum dots (GQDs), carbon quantum dots (CQDs), and carbonized polymer dots (CPDs). These nanostructures are attractive because they are stable, simple to prepare, diverse, and have excellent water solubility. Drug delivery, cancer imaging and treatment, bioimaging, and sensing are just a few areas where their tiny size, unique optical characteristics, and excellent biocompatibility have gained a lot of interest in biomedical research [11]. Zero-dimensional materials QDs have unique opto-electronic capability and fluorescent properties with an extensible band gap energy (2.2 to 3.1 eV), and they can be actively coupled with other materials, which enhances their sensitivity in sensor applications [12]. Carbonaceous nanomaterials are often synthesized from biomass, which is affordable, nontoxic, and available. Cocoon silk, a biomass from Bombyx mori silkworms, is of great importance due to its silk fibroin content, which may be readily dissolved for regeneration into diverse morphologies [13].

Synthetic polyamidoamine (PAMAM) dendrimers have a tree-like structure and a homogeneous nanometric size range of 3–6 nm. PAMAM dendrimers may be surface functionalized via chemical conjugation or electrostatic interactions due to their free amine and carboxyl groups [14]. In its fourth generation, the molecule has 64 amine groups on its surface for bioreceptor coupling. Numerous dendrimer-enzyme combinations have been used in electrochemical biosensors, primarily in glucose oxidase-based glucose sensors. The nanomaterials discussed above are predicted to enhance electron transfer and provide electrocatalytic advantages [15].

The tTG was covalently attached to the electrode surface using carbodiimide conjugation chemistry, allowing for controlled immunorecognition by autoantibodies and rapid detection of anti-tTG antibodies, through electrochemical diffusion. Electrochemical detection is a simple, affordable, and user-friendly analytical approach presented in this article. Our approach combines the benefits of immunochemical tests with electrochemical transduction, without a secondary enzyme-labeled antibody.

## 2. Materials and Methods

### 2.1. Preparation of the QDs Fluorescent Nanostructures

The fluorescent QDs were made using silk fibroin as a precursor using the hydrothermal method described by Qun Wang and coworkers, with small modifications [13]. A microfarm in Stoenesti, Valcea, Romania, produced silk butterfly cocoons. We chopped the cocoons into small pieces after removing the larvae. We boiled chopped cocoons in a 0.02 M sodium carbonate solution (Na_2_CO_3_, ACS reagent, anhydrous, ≥99.5%, Sigma Aldrich) for 30 min to get sericin out of the silk thread. The following step consisted of washing the obtained fibroin to remove excess Na_2_CO_3_ and then drying it at room temperature. Next, 4 mL of LiBr (≥99.995% trace metal base, Sigma Aldrich, Saint Louis, MO, USA) 9.3 M solution was added drop-by-drop to 1 g of dried fibroin to dissolve. We performed the operation at 60 °C in an oven until the color turned amber and all the fibers melted. We syringed the dissolved fibroin solution into a dialysis bag (cellulose membrane, average flat width 33 mm, Merk, Darmstadt, Germany), and performed the dialysis process in ultrapure water for 48 h, necessary for the removal of salt. The magnetic stirring dialysis at ambient temperature involved at least six water changes. The fibrin solution’s calculated concentration was 7.5% by weight. A 50 mL hydrothermal synthesis autoclave reactor with a PTFE-lined vessel (Cambridge Energy Solutions, Cambridge, UK) was filled with 30 mL of dialyzed fibroin solution. It was then put in an electric oven set to 220 °C and left there for 12 h. To eliminate large granules, we centrifuged the solution (benchtop centrifuge, ROTINA 380 R, Hettich, Beverly, MA, USA) at 10,000 RPM for 30 min in the final step. We named the solution QDsSF and stored it at 4 °C for future use.

### 2.2. Preparation of the Modified Electrodes

We used 3 mm glassy carbon electrodes (GC, Metrohm, Schiedam, The Netherlands) in this study. First, we polished them using the Metrohm polishing kit with alumina (Metrohm, Schiedam, The Netherlands), and then, to obtain mirror surfaces, we used monocrystalline diamond paste (1/4 μ) on TFR polishing cloths (PRESI, Eybens, France). Electrochemical modification was carried out in a three-electrode electrochemical cell (Metrohm, Schiedam, The Netherlands). The working electrode was GC, the reference electrode was an Ag/AgCl 3M KCl (from Metrohm, Schiedam, The Netherlands), and the counter electrode was a Pt rod (from Metrohm, Schiedam, The Netherlands). We conducted the electrochemical experiments at room temperature and under daylight using an Autolab Potentiostat/Galvanostat PGSTAT 302N (Metrohm, Schiedam, The Netherlands).

The GC/PPy electrode’s electrolyte solution is prepared by dissolving 0.7 g of pyrrole monomer (98% Alpha Aesar, Thermo Fisher, Kandel, Germany) and 0.98 g of paratoluene sulphonic acid (TsOH, monohydrate, 97% from Thermo Fisher Scientific, Heysham, UK) in 50 milliliters of distilled water. To prepare GC/PPy-QDsSF, we added 0.5 mL of QDsSF solution to the electrolyte described before. The electropolymerization of both electrodes was carried out using chronoamperometry at a voltage of 0.85 V and a cut-off current of 1 mC. We rinsed the electrodes with distilled water.

To prepare the GC/PPy-QDsSF-PAMAM electrode the aqueous solution was made with 0.4 M N-(3-dimethylaminopropyl)-N′-ethylcarbodiimide hydrochloride (EDC, commercial grade, powder, Sigma Aldrich, Saint Louis, MO, USA) and 0.1 M N-hydroxy succinimide (NHS, 98%, Sigma Aldrich, Saint Louis, MO, USA). 10 μL of PAMAM dendrimer (ethylenediamine core, 10 wt% solution in methanol, generation 4.0, Sigma-Aldrich, Saint Louis, MO, USA) was mixed with 1 mL of the solution. The solution was mixed with a vortex (DLAB-MX-S, DLAB Scientific, Beijing, China), and 30 μL were pipetted (using Eppendorf Research Plus micropipette, Ependorf, Wien, Austria) on the GC/PPy TsOH-QDs SF electrode. It was then left to react at room temperature for one hour.

0.1 M PBS pH 7.4 from a 0.2 M stock solution of sodium phosphate monobasic (NaH_2_PO_4_) (anhydrous, approximately 99%, Sigma Aldrich, Saint Louis, MO, USA) and sodium phosphate dibasic (Na_2_HPO_4_) (puris, 98–100%, Sigma Aldrich, Saint Louis, MO, USA) were prepared. 10 μg of recombinant human transglutaminase 2 protein (tTG) (1 mg/mL, ABCAM Limited, Cambridge, UK) was mixed with 500 μL of 0.1 M PBS pH 7.4 to obtain Solution 1. Another aqueous solution, named Solution 2, was prepared with 0.2 M EDC and 52 mM N10 μL of Solution 1 and 20 μL of Solution 2 were mixed thoroughly in an Eppendorf tube using a Vortex mixer. They were then put on a GC/PPy-QDsSF-PAMAM electrode using an Eppendorf micropipette and left to rest at room temperature for one hour to help the -COOH groups of tTG and the -NH_2_ groups of PAMAM form an amide bond. After washing with a 0.1 M PBS pH 7.4 solution, we removed the unbound antigen (tTG) was removed. Before use, we allowed the GC/PPy-QDsSF-PAMAM-tTG electrode was left to dry at room temperature.

### 2.3. QDsSF Solution Characterization

Spectra of the QDsSF solution were obtained. The UV-VIS spectra were recorded using a Perkin-Elmer L950 UV-Vis/NIR spectrophotometer (Perkin Elmer, Shelton, CT, USA). It was used a Perkin-Elmer Spectrum 100 to take FT-IR spectra between 4000 and 600 cm^−1^. Four successive scans with a resolution of 4 cm^−1^ were registered. The fluorescence spectra at room temperature were recorded using a JASCO FP-6500 Spectrofluorometer (Jasco, Tokyo, Japan) and a quartz cuvette with a path length of 1 cm. The excitation and emission silts were set at 10 and 5 nm. At least three determinations were replicated.

### 2.4. Surface Characterizations

Modified electrodes surface morphology was observed with an Inspect F50 scanning electron microscope and energy-dispersive spectrometer (EDS) (Thermo Fisher Scientific, formerly FEI, Eindhoven, The Netherlands). GC electrodes were fixed and placed into the microscope analysis chamber using a carbon-bearing strip. They were coated with a thin gold coating to minimize electric charges.

FT-IR spectra were also recorded for modified electrodes using the same equipment and conditions as mentioned for QDs solutions.

For band gap determination, a Double Beam UV-Visible/NIR Spectrophotometer (Jasco, Tokyo, Japan) was used. Reflectance spectra were recorded between 200–850 nm. The bandgap (Eg), of the samples was determined by using the Kubelka-Munk method to diffuse reflectance spectra. The band gaps associated with the direct transitions of the samples were determined by extrapolating the linear portion of the plot of (αhυ)^2^ versus (hυ) using The Kubelka-Munk or remission function, as described in literature [16,17]. Determinations were performed only for GC/PPy sample and GC/PPy-QDsSf sample to see the quantum dots influence on polypyrrole film.

### 2.5. Electrochemical Characterizations 

Three-electrode electrochemical cells (Metrohm, Schiedam, The Netherlands) were used for all electrochemical characterisation tests. The reference electrode was an Ag/AgCl in 3 M KCl (from Metrohm, Schiedam, The Netherlands), the counter electrode was a Pt rod, and the working electrode was GC or modified GC. An Autolab Potenti-ostat/Galvanostat PGSTAT 302N (Metrohm, Schiedam, The Netherlands) was used for ambient and daylight experiments. Electrolyte used was 0.1 M PBS pH 7.4 solution or 0.1 M PBS pH 7.4 with 5 mM potassium ferrocyanide K_4_[Fe(CN)_6_] (ACS reagent, 98.5–102.0%, Sigma Aldrich, Saint Louis, MO, USA) and 5 mM potassium ferricyanide K_3_[Fe(CN_6_)] (99%, Sigma Aldirch, Saint Louis, MO, USA).

Electrochemical impedance spectroscopy (EIS) values at the open circuit potential were 3700 to 0.27 Hz and 0.01 V.

For cyclic voltammetry (CV), the voltage was changed from −0.2 to 0.7 V or −0.1 to 0.6 V (against Ag/AgCl, 3 M KCl) with a 2 mV step and 50 mV/s scan rate. CV recordings were also made at 20–200 mV/s.

Sware wave voltammetry (SWV) measurements were performed between −0.1 and 0.8 V (vs. Ag/AgCl, 3 M KCl reference electrode) with 5 mV step, 80 mV amplitude, at 10 Hz frequency.

The stability of the GC/PPy-QDsSF-PAMAM-tTG electrode was investigated by measuring the current response during 100 consecutive CV scans in 0.1 M PBS pH 7.4 with Fe^2+^/Fe^3+^ redox couple with a 50 mV/s scan rate.

The stability over a range of extended time periods was also tested by preparing nine identical GC/PPy-QDsSF-PAMAM-tTG electrodes. Three electrodes were scanned immediately after preparation, followed by three more after 20 days, and the remaining three after 30 days. Those scanned after 20 or 30 days were kept in the fridge at 4 °C. Scans were performed by SWV in 0.1 M PBS pH 7.4 with 5 mM K_4_[Fe(CN)_6_] and 5 mM K_3_[Fe(CN_6_)].

GC/PPy-QDsSF-PAMAM-tTG electrodes were created to study how various blood components affect electrode signal. Three samples were incubated with 30 μL of Elisa kit IgA 0 U/mL anti-tissue antibody standard solution for 1 h at room temperature. On the other electrodes surface, 30 μL was pipetted from the following solutions using a micropipette: 4 g/dL bovine serum albumin, 2.5 g/dL γ globulin, and 0.9 mg/mL creatinine. Albumine, creatinine and γ globulin were prepared in 0.1 M PBS pH 7.4. After incubating for one hour at room temperature, incubated electrodes were rinsed with PBS and the SWV curves were recorded in 0.1 M PBS pH 7.4 with 5 mM K_4_[Fe(CN)_6_] and 5 mM K_3_[Fe(CN_6_)].

### 2.6. Calibration Curve and Real Sample Analysis

Freshly prepared GC/PPy-QDsSF-PAMAM-tTG electrodes were incubated for 1 h with 30 μL commercial standardized IgA antibodies solutions (ORGENTEC Diagnostika GmbH, Mainz, Germany) then washed with PBS. A calibration curve was obtained using SWV (same parameters as mentioned at Section 2.5) performed with the same equipment, electrodes and cell (as mentioned at Section 2.2) recorded in PBS pH 7.4 with 5 mM K_4_[Fe(CN)_6_] and 5 mM K_3_[Fe(CN_6_)]. Measurements were made in triplicate.

Real samples were analyzed with the modified electrode GC/PPy-QDsSF-PAMAM-tTG electrodes, after 1 h incubation with 30 μL patient sample followed by rinsing with PBS and SWV in PBS pH 7.4 with 5 mM K_4_[Fe(CN)_6_] and 5 mM K_3_[Fe(CN_6_)]. Real samples were analyzed also with an enzyme-linked immunosorbent assay (ELISA) with calibration curve and human recombinant tissue transglutaminase coated microwells was used as recommended by the ESPGHAN celiac disease diagnosis guidelines [18]. The cut-off for positivity (upper limit of normal) was set at 10 U/mL as recommended by the kit producer (ORGENTEC Diagnostika GmbH, Mainz, Germany). Obtained results were compared with SPSS software, version 30.0.0 and sensitivity, specificity, positive and negative predictive value were calculated for the newly proposed method.

## 3. Results and Discussion

### 3.1. Spectral Characteristics of Prepared QDsSF

**Figure 1:**
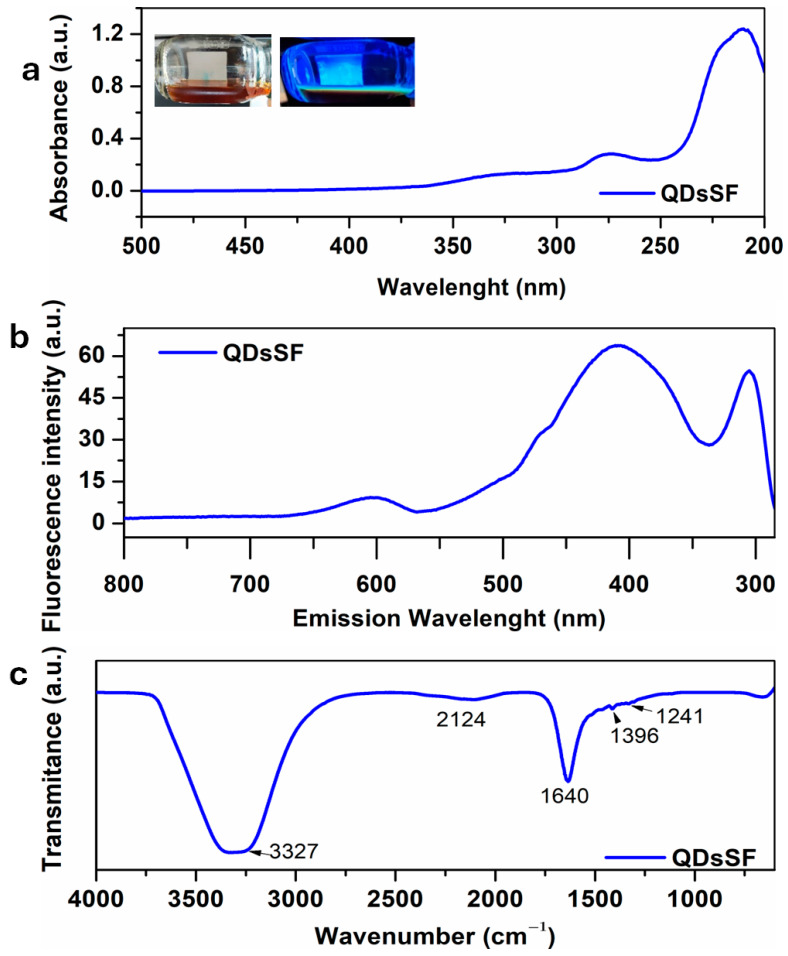
Spectra for QdsSF solution: (**a**) UV-Vis absorption, (**b**) Florescence, (**c**) FT-IR.

Prepared QDs were spectroscopically characterized, and the obtained results are presented in Figure 1. UV-Vis absorption spectra of the QDsSF can be seen in Figure 1a. They exhibited a strong absorption in the UV region, extending into the visible region. A broad peak at 220 nm, caused by a p-p* transition, points to the presence of functional groups like carboxyl groups and other electron pairs that are not connected to anything else in the QDsSF. Additionally, a small absorption peak appears at 276 nm. In day light, the QDsSF solution is brown, and under a 365 nm UV lamp it exhibited yellow-green fluorescence (as shown in the inset of Figure 1a inset) [19].

The emission of light or other radiation from a material that has absorbed light or other electromagnetic radiation is known as fluorescence. This physical process involves the energy absorption by the substance, followed by re-radiation at longer wavelengths than the incident light; multiphoton absorption allows the re-radiation at a shorter wavelength. Fluorescence is a prevalent form of luminescence found in nature and widely used in practical applications. The most attractive feature of fluorescent silk materials is their ability to fluoresce under invisible light, such as UV light, and display a range of vivid colors visible to the human eye [20]. Fluorescence QDs typically exhibit best characteristics in aqueous phase at low concentrations, while concentrated QDs often experience aggregation-induced quenching (AIQ) via π-π intermolecular interactions [21], so recordings were made using diluted solution.

The fluorescence spectra of the QDsSF is shown in Figure 1b. The QDsSF solution has a relatively symmetrical excitation spectrum, with a maximum excitation wavelength of 420 nm at 275 nm excitation wavelength. This indicates the presence of QDs obtained from silk fibroin.

For a complete investigation of the surface functional groups and structural components of the synthesized QDs, it was performed infrared spectroscopy characterization. Figure 1c presents the FT-IR spectra of the synthesized QDsSF. In the FT-IR spectrum, two prominent absorption peaks can be observed at 1640 cm^−1^ and 1396 cm^−1^, corresponding to the stretching vibrations of C=O and C–N, respectively. The peak at 3327 cm^−1^ is associated with the stretching vibration of C–O in –OH and unsaturated hydroxyl groups, indicating the presence of both carboxyl and hydroxyl groups on the surface of the synthesized QDs [19].

### 3.2. Surface Characterizations of Modified Electrodes

**Figure 2:**
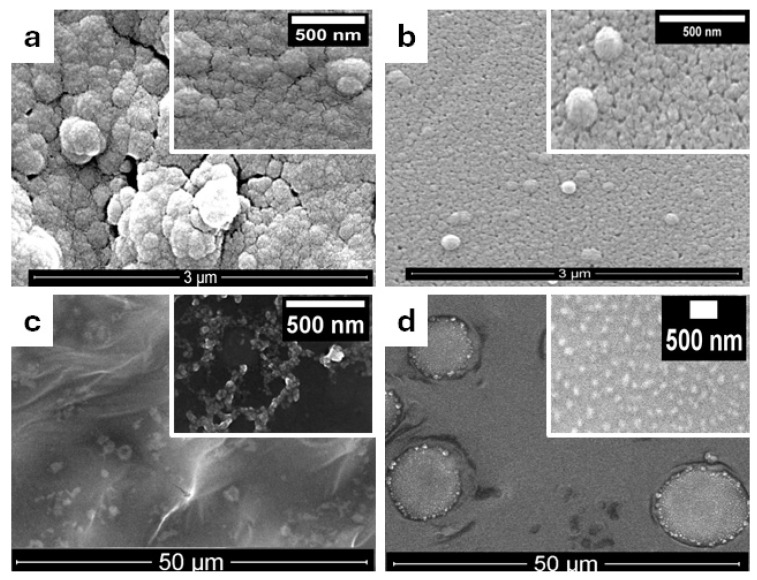
SEM images of modified electrodes after each step, at different magnifications: (**a**) GC/PPy, (**b**) GC/PPy-QDsSF, (**c**) GC/PPy-QDsSF-PAMAM, (**d**) GC/PPy-QDsSF-PAMAM-tTG.

To verify the morphology, typical top view SEM images for the modified electrodes are depicted in Figure 2. TsOH was employed as the “soft” template in a one-step electrochemical polymerization of pyrrole onto the polished GC surface, resulting in the development of ordered PPy, a uniform, and porous nanostructured layer visible in Figure 2a. The grains have a roughly spherical shape and intertwine to create cohesive, cauliflower-like formations that coil in a regular pattern, resulting in a three-dimensional arrangement. The globules exhibited isotropy and displayed interconnections at diverse angles. When QDs are added to polymerization solution (Figure 1b), there are no important morphology changes compared to GC/PPy. The dendrimer PAMAM’s attachment is confirmed by the presence of a small globular polymeric structure on the GC/PPy-QDsSF-PAMAM electrode surface (Figure 1c inset). The compact globular polymeric structure of PAMAM is no longer visible on the GC/PPy-QDsSF-PAMAM-tTG electrode surface in Figure 2d, where it is likely hidden by the tTG enzyme.

**Figure 3:**
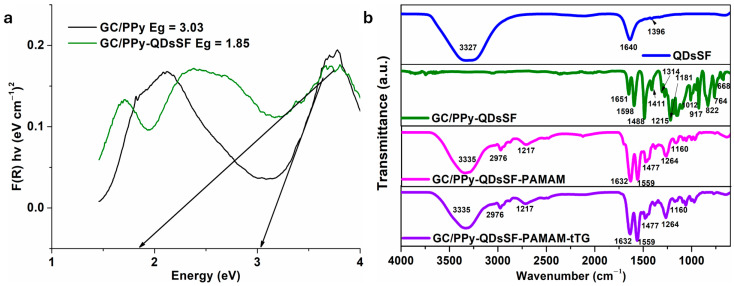
(**a**) Kubelka-Munk transformed reflectance spectra of GC/PPy (black line) and GC/PPy-QDsSf (olive line); (**b**) FT-IR images corresponding to the samples: QDsSF (blue line); GC/PPy-QDsSF (olive line); GC/PPy-QDsSF-PAMAM (magenta line) and GC/PPy-QDsSF-PAMAM-tTG (violet line).

To see the influence of QDs on polypyrrole film, we determined the optical band gap for GC/PPy si and GC/PPyQDsSF samples was determined. The graph is presented in Figure 3a. The detected band gap (Eg) of the samples varied between 3 eV for GC/PPy and 1.85 eV for GC/PPyQDsSF, which are much less significally lower than the band gap of TiO_2_ (3.20 eV) for example. The observed phenomenon may be attributed to the creation of a bipolaron band, which is enhanced by the introduction of doping [22], in our case with quantum dots (QDs). Results are consistent in agreement with other research findings described in literature for PPy [17,22]. Lower band gap indicates that the polymer nanostructures may be activated by visible light, making them potentially useful in optoelectronics, photocatalysis, or electrocatalysis [17].

Figure 3b shows the FITR spectra of the QDsSF, GC/PPy-QDsSF, GC/PPy-QDsSF-PAMAM and GC/PPy-QDsSF-PAMAM-tTG nanocomposites. The FTIR spectra of GC/PPy-QDsSF exhibit characteristic peaks at 668 and 764 cm^−1^, corresponding to the C–H wagging out-of-plane deformational vibration mode of the PPy ring. Peaks around 764–1181 cm^−1^ represent the C–H in-plane and out-of-plane deformation in PPy units. The peak at 917 cm^−1^ corresponds to the C=N^+^−C stretching vibration. The peaks at 1488 cm^−1^ and 1598 cm^−1^ are attributed to the vibrations of the pyrrole ring and C=C stretching vibrations. Peaks at 1314, 1181, and 1012 cm^−1^ are related to C=N bending, C−N stretching, and =C−H bending vibrations of PPy, respectively [23,24,25]. This shows that the PPY PPy was successfully attached to the electrode surface.

For GC/PPy-QDsSF-PAMAM, a strong peak at 3335 cm^−1^ may originate from the absorption assignable to loaded PAMAM in PPy or the interactions at the interface of PAMAM and PPy. The FTIR spectrum of blank PAMAM dendrimers shows peaks at 3335 cm^−1^ and 2976 cm^−1^, indicating the presence of terminal primary amino groups. The peaks at 1559 cm^−1^ and 1632 cm^−1^ correspond to N–H bending/C–N stretching (amide II) and C–O stretching (amide I) vibrations, respectively [26,27]. These bands overlap with the characteristic peaks of PPy. Additionally, the positions of the peaks in the GC/PPy-QDsSF-PAMAM nanocomposite spectra are shifted to higher wavenumbers compared to the GC/PPy-QDsSF, likely due to the compound formed between GC/PPy-QDsSF and PAMAM.

By adding the anti-tissue transglutaminase (tTG) enzyme, the FT-IR spectrum does not change.

### 3.3. Electrochemical Characterizations of Modified Electrodes

**Figure 4:**
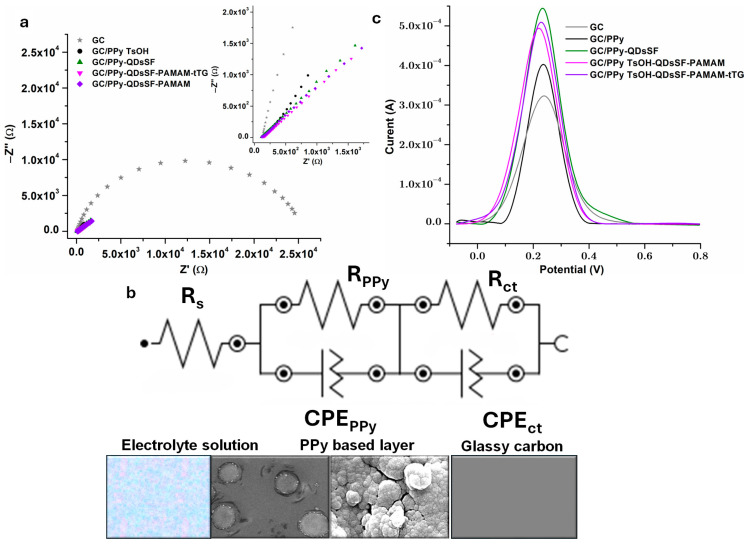
(**a**) EIS for modified electrodes (Nyquist diagram) recorded vs. Ag/AgCl 3 M KCl in PBS + Fe^2+^/Fe^3+^ (**b**) equivalent circuit to fit the data, (**c**) SWV curves corresponding to modified electrodes vs. Ag/AgCl 3 M KCl in PBS + Fe^2+^/Fe^3+^.

The characteristics of the PPy based films/ PBS electrolyte interface were examined using electrochemical impedance spectroscopy (EIS), measurements conducted at the Open Circuit Potential, as shown in Figure 4a. The circuit for the polished GC electrode was composed of the PBS solution resistance (Rs) coupled in series with a second circuit representing charge transfer at the GC/PBS solution interface. This second circuit consists of a resistor (Rct) in parallel with a constant phase element (CPEct). For modified electrodes, the circuit seen in Figure 4b, becomes more complex. It has Rs in series with a second circuit representing charge transfer at the GC/PPy interface and includes an extra combination of a resistor (R_PPy_) and a constant phase element (CPE_PPy_) that corresponds to the ionic charge-transfer resistance at the conducting polymer-PBS electrolyte interface.

The most likely electrical equivalent circuit for the tested samples was determined by fitting EIS data using NOVA software, version 1.11; Table 1 presents the obtained values. Table 1 shows that Rs values are consistent across all tasting samples, with values around 100 Ω, because the same PBS solution was used for testing. A pseudo-capacitive behavior is indicated by Nct values above 0.5 for most samples. Rct, the resistance ascribed to charge transfer at the interface between the GC and PBS solutions, has values in the kilo Ohms range, with the maximum value for GC and GC/PPy samples and the lowest value for GC/PPy-QDs sample. For every electrode having PPy-QDsSF layer, R_ct_ is about the same.

The RPPy value for polypyrrole coatings is hundreds of Ohms, lower than the Rct values. This suggests that the coatings do not create a homogeneous, insulating layer as shown in SEM pictures. The pseudo-resistance of this layer is characterized by low NPPy values, in 0.35–0.5 domain.

The Chi-square value varied from 0.006 to 0.045, suggesting strong agreement between the comparable circuit and data.

Figure 4c shows the SWV curves recorded in PBS with redox couple after each modification step. All samples gave a peak at around 0.22 V. For all modified electrodes, the signal is higher compared to the one for GC, suggesting an improved conductivity. As it was expected after band gap measurements, QDsSF presence in PPy film led to an increase in the signal. The presence of tTG enzyme immobilized with a cross-linker on the surface decreases the current. It is known that bioactive chemicals may restrict electrochemical probe (Fe^2+^/Fe^3+^) accessibility and impede interfacial electron transport [28].

**Table 1:** Parameters obtained from EIS data fitting using Nova software 1.11.

	Parameter	Rs (Ω·cm^2^)	Rct (Ω·cm^2^)	CPEct		RPPy (Ω·cm^2^)	CPE_PPy_		χ2
Sample *				Yoct (S·sn)	Nct		YoPPy (S·sn)	NPPy	
GC/PPy-QDsSF		109.04	0.61 × 10^4^	32.2 × 10^−4^	0.71	397.31	19.6 × 10^−4^	0.52	0.016
GC/PPy-QDsSF-PAMAM		106.61	0.82 × 10^4^	22.8 × 10^−4^	0.59	112.2	24.8 × 10^−4^	0.35	0.023
GC/PPy-QDsSF-PAMAM-tTG		139.28	0.74 × 10^4^	26.6 × 10^−4^	0.61	221.7	30.3 × 10^−4^	0.40	0.045

* for GC and GC/PPy electrodes see data from [29].

**Figure 5:**
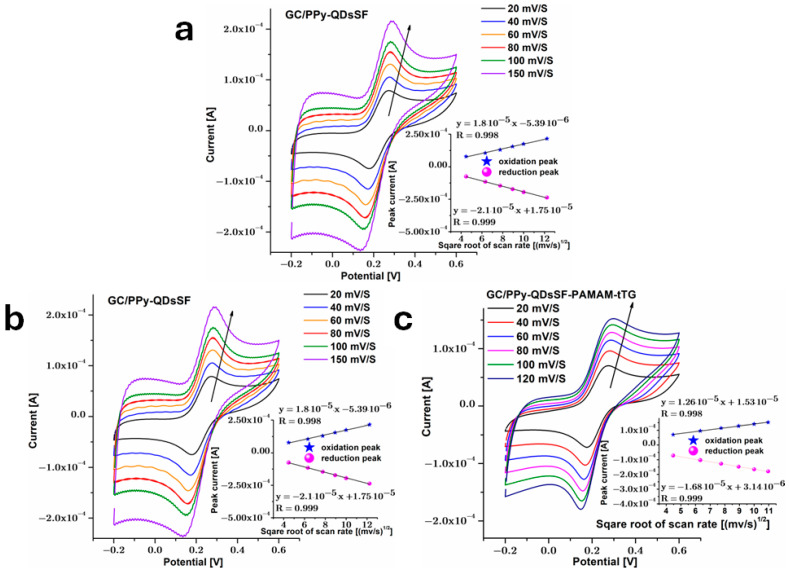
CVs (2nd cycle) recorded in PBS with Fe^2+^/Fe^3+^ vs. Ag/AgCl 3M KCl, at different scan rates corresponding to (**a**) GC/PPyQDsSF, (**b**) GC/PPy-QDsSF-PAMAM and (**c**) GC/PPy-QDsSF-PAMAM-tTG.

Having PBS as a supporting electrolyte and equal concentrations of Fe^2+^ and Fe^3+^, a cyclic voltammetry curve was recorded at different scan rates for modified electrodes, and the obtained results are presented in Figure 5. These were recorded starting from the premises that controlled dispersion of [Fe(CN)6]^3−/4−^ on surfaces without adsorption is essential for electrochemical biosensors [30]. For each sample, the anodic and cathodic peak currents of the Fe^2+^/Fe^3+^ redox reaction rise linearly with scan rate. The findings shown in Figure 5 indicate that the transfer of electrons between the [Fe(CN)6]^3−/4−^ solution and the electrode occurs at a faster rate compared to the migration of electroactive species from the bulk solution to the electrode interface, which is driven by the concentration gradient [31].

Theoretical studies have shown that redox reactions exhibit surface processes at low scanning rates on modified electrodes, and the behavior transitions towards diffusion control when the sweep rate is raised. Therefore, it can be inferred that the kinetics of the Fe^2+^/Fe^3+^ redox process is governed by diffusion [32]. The connection between the diffusion current (Ip) and the process may be described by the Randles-Sevcik [33] Equation (1) as follows:

(1) $$I_{p}=2.69\cdot 10^{5}An^{3/2}CD^{1/2}\nu^{1/2}$$

where: A is the area of the GC electrode, n = 1 (the number of electrons), C is the concentration in mol/cm^3^ of Fe^2+^ or Fe^3+^ (5 mM for this study), D is the diffusion coefficient and ν is the scan rate (100 mV/s). The value of diffusion coefficient D was determined by making CV scans at different scan rates for a GC bare electrode with a geometrical area of 0.07 cm^2^. Then the active area of modified electrodes was calculated: 0.104 cm^2^ for GC/PPy-QDsSF; 0.086 cm^2^ for GC/PPy-QDsSF-PAMAM and 0.084 cm^2^ for GC/PPy-QDsSF-PAMAM-tTG. It is visible from SEM images that tTG enzyme reduces surface porosity forming a film on electrode surface.

### 3.4. Modified Electrode Repeatability, Stability and Behavior in Time

Figure S1a presents the results for electrode repeatability. The obtained signal clearly shows similarities. Excel software version 2411 revealed an RSD of 0.06%, indicating the repeatability of the modified electrode preparation.

The result of the stability test during 100 consecutive CV scans is displayed in Figure S1b. In repeated testing, this electrode showed good repeatability and no surface fouling during voltametric measurements.

Figure S1c displays the results of stability for 30 days. We had an average signal loss of about 16% after 20 days of fridge storage for GC/PPy-QDsSF-PAMAM-tTG electrode and after 30 days, it is 18%, so the modified electrode exhibits adequate stability and activity over 30 days. The minor reduction may be ascribed to the antigen’s inactivation over time. A possible explanation for good behavior of modified electrode could be the robust interactions between tTG and PPy-QDsSF.

The specificity of the modified electrode incorporating anti-tTG antibody and several non-specific serum proteins (albumin, creatinine, γ-globulin) was evaluated by SWV measurements and the results are presented in Figure S1d. The obtained signal shows little influence from the tested proteins. The modified electrode’s SWV peak-current in non-specific serum proteins was like or slightly lower than the signal obtained for GC/PPy-QDsSF-PAMAM-tTG electrode with 0 U/mL antibody. It is evident that the signal is decreasing only in the presence of tTG antibodies, confirming what it was known form literature that autoantibodies produced in CD patients selectively target tissue transglutaminase (tTG) protein [1].

### 3.5. Antibodies Presence Determination with Modified Electrode

**Figure 6:**
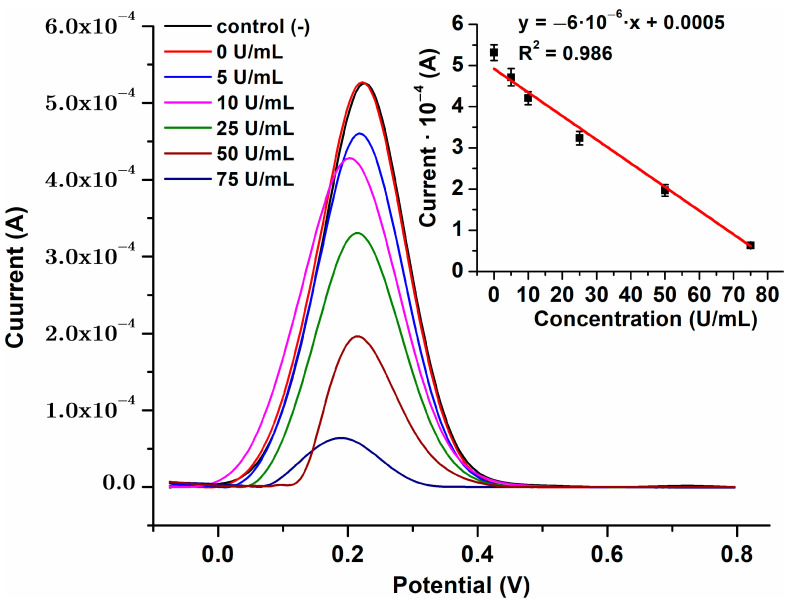
Calibration curve using GC/PPy-QDsSF-PAMAM-tTG electrode and standard ready to use anti tissue antibody solution.

Identical GC electrodes were freshly modified. Calibration curve was recorded with standard ready to use solutions provided with Elisa test kit. For each point on the curve, experiments were performed in triplicate and the average values were used for each antibody concentration. Using Excel, we also determined standard deviation for each case. The obtained results can be seen in Figure 6. In the absence of anti-tissue transglutaminase antibodies, the modified electrode exhibits a greater current response. Conversely, the presence of anti-Tissue transglutaminase antibodies results in a reduction of peak current in SWV as the concentrations of these antibodies in serum grow. This resulted from the increased development of antigen-antibody complexes on the working electrode surface, leading to a reduction in redox current. It is visible that we obtained a good linearity between 0 and 75 U/mL anti-Tissue transglutaminase antibodies, with R^2^ = 0.986.

A linear regression analysis on obtained calibration curve data was performed using Excel’s built-in “Data Analysis”. Using the standard deviation (SD) of response (σ) and slope (s) of the calibration curve obtained in Excel, the LOD was determined using formula [3.3·(σ/s)] described in literature [34]. For this study, LOD was found to be equal to 7.56 U/mL, below the threshold value of 10 U/mL established as reference for CD disease.

Recent reports indicate the application of electrochemical detection methods for detecting autoantibodies specific to coeliac disease, primarily anti-tTG antibodies, employing various surface chemistry methodologies. Compared with other results reported in the literature (Table 2) for electrochemical detection of CD specific antibodies, the proposed modified electrode and the method used in this study (SWV) gave comparable results. In our case the detection limit is a little higher compared to other reported cases, but all the patients with CD have elevated levels of antibody, above 10 U/mL. Although detection was not possible for antibody values above 80 U/mL, as in the case of the commercial Elisa kit, the proposed modified electrode can discriminate between healthy patients and those with celiac disease.

**Figure 7:**
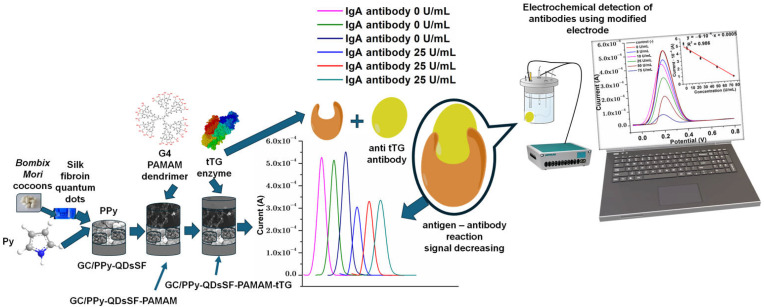
Schematic illustration for preparation of GC/PPy-QDsSF-PAMAM-tTG electrode and the detection strategy for anti-tissue antibody detection.

The proposed modified electrode used in this study was prepared based on a layer-by-layer approach as illustrated in Figure 7. Glassy carbon (GC), whose benefits are its extensive surface area, superior electrical conductivity, low charge transfer resistance, electrochemical inertness throughout an extensive potential range, elevated hardness, chemical stability, impermeability, and facilitation of surface modification [42,43], was chosen as substrate. Polypyrrole was used because exhibits notable characteristics, including strong biocompatibility, elevated electronic conductivity, ease of preparation, and substantial environmental stability [44]. Among the various dopants for PPy, p-toluenesulfonic acid (p-TS) was selected for its performance, water solubility, and ability to modify the structure and morphology of the conducting polymer, thereby enhancing conductivity, carrier mobility, chemical stability, crystallinity, thermoelectric properties, and charge transfer capabilities [45]. But the covalent immobilization of biomolecules on pure PPy film is challenging due to the lack of functional groups, including carboxyl, hydroxyl, or amino groups [44]. To overcome this, quantum dots were used for their high surface-to-volume ratio, multiple molecule recognition sites, biocompatibility, low cytotoxicity, water solubility, potential for surface hydrophilicity enhancement, and stable photoluminescence [46,47,48]. Bombyx mori silk, characterized by a nitrogen content of up to 18%, has been utilized as a raw material for the preparation of these nanostructures [19] via a hydrothermal method. The prepared GC/PPy-QDsSF has showed a narrowed band gap from 3.03 to 1.85.

Further, the remarkable attributes of quantum dots were integrated with those of PAMAM. Poly (amidoamine) is utilized owing to its branched, tree-like polymeric structure, which possesses a substantial quantity of amino groups for conjugation with targeted carboxylate probes [49]. tTG acts as the capture element that binds the target anti-tTG, as these autoantibodies produced in persons with CD specifically target the tissue transglutaminase (tTG) protein [1].

70 real patients’ samples were analyzed with the proposed modified electrode. Using calibration curve, antibody level was calculated for each of them. We used an established limit of 10 U/mL to divide the patients into positive and negative. In blind, in parallel, patient samples were tested with TG2-IgA an enzyme-linked immunosorbent assay. Results were compared using SPSS and Excel software. 18 of these patients were true positive, 24 true negative, 14 false negative and 14 false positive. A 56.3% sensitivity and 63.16% specificity were obtained. The positive predicted value was 56.3% and negative predictive value was 63.16%.

## 4. Conclusions

SEM images reveal the deposition of an uniform and porous nanostructured PPy layer on the GC electrode. Band gap measurements revealed that the hydrothermally prepared quantum dots from silk fibroin can act as dopants for PPy and decrease the band gap. We successfully attached the G4 PAMAM dendrimer to the electrode surface using NHS/EDC cross-linkers. We also grafted the tissue transglutaminase enzyme onto the electrode surface to enable the detection of anti-tissue antibodies. SEM and FT-IR analysis confirmed the surface modification.

The proposed GC/PPy-QdsSF-PAMAM-tTG electrode has less resistance and more surface area, as shown by impedance and CV measurements.

Additional tests showed that the modified GC/PPy-QdsSF-PAMAM-tTG electrode is stable during consecutive scans and over time, repeatable when scanned in PBS with redox couple solution, and the specific detection of anti-tissue antibodies is unaffected by the presence of albumin, creatinine, or globulin.

With a detection limit of 7.56 U/mL, the modified electrode can detect anti-tissue antibodies up to 80 U/mL and has the potential to be used in a real-world setting to analyze human blood serum samples.

## Figures and Tables

**Table 2:** Comparison of anti-tTG antibodies linear domain and limit of detection obtained using electrochemical methods.

Used for Detection	Linear Domain	Limit of Detection	Reference
screen-printed modified with CdSe/ZnS QDs	0–40 U/mL	2.2 U/mL	[1]
screen-printed carbon electrodes modified with * ^1^ MWCNTs and gold np.	0–40 U/mL	not mentioned	[35]
disulfide-modified antigen	0.26–6.9 μg/mL	0.26 μg/mL	[36]
8-channel screen-printed electrodes and anti-human IgA labelled with biotin and streptavidin labelled with CdSe/ZnS quantum dots	0–40 U/mL	2.7 U/mL	[37]
* ^2^ GC/Au/MUA functionalized electrodes	0–30 U/mL	1.7 U/mL	[38]
gold nanoelectrode and polycarbonate track-etched membranes	0.005–1.0 µg/mL	1.8 ng/mL	[39]
gold deposited on track-etched polycarbonate	0.25–8.54 U/mL	0.7 U/mL	[40]
DNA oligomer anchored to a gold electrode surface	0.01–10 U/mL	~10^−2^ U/mL	[41]
GC/PPy-QDsSF-PAMAM-tTG	0–75 U/mL	7.56 U/mL	This work

* ^1^ MWCNTs—multiwalled carbon nanotubes. ^2^ GC/Au/MUA—glassy carbon/nanogold substrate/11- mercaptoundecanoic acid.

## Data Availability

The original contributions presented in this study are included in the article/Supplementary Materials. Further inquiries can be directed to the corresponding author(s).

## Supplementary Materials

The following supporting information can be downloaded at: https://www.mdpi.com/article/10.3390/bios15010042/s1.
