# Supplementary material for: Anti-Tissue-Transglutaminase IgA Antibodies Presence Determination Using Electrochemical Square Wave Voltammetry and Modified Electrodes Based on Polypyrrole and Quantum Dots
Source: Biosensors (Basel). 2025 Jan 13;15(1):42. doi: 10.3390/bios15010042 (PMC11764343; doi:10.3390/bios15010042)
Supplement: Supplementary file 1 [file biosensors-15-00042-s001.zip › biosensors-3368385-supplementary.pdf]

Supplementary materials

Modified electrode repeatability, stability and behavior in time

Five freshly prepared GC/PPy-QDsSF-PAMAM-tTG electrodes were subjected to SWV measurements in 0.1 M PBS pH 7.4 with Fe^{2+}/Fe^{3+} redox couple. Fig. S1 a present the results.

The result of the stability test is displayed in Fig. S1 b. It is apparent visible that the obtained signal is stable during 100 consecutive CV scans in 0.1 M PBS pH 7.4 with Fe^{2+}/Fe^{3+} redox couple as anodic and cathodic peak currents remained almost constant.

Fig. S1 c displays the results of stability measurements for GC/PPy-QDsSF-PAMAM-tTG electrode for 30 days.

Fig. S1 d presents the results of SWV curves recorded in 0.1 M PBS pH 7.4 with 5 mM $K_4[Fe(CN)_6]$ and 5 mM $K_3[Fe(CN_6)]$.

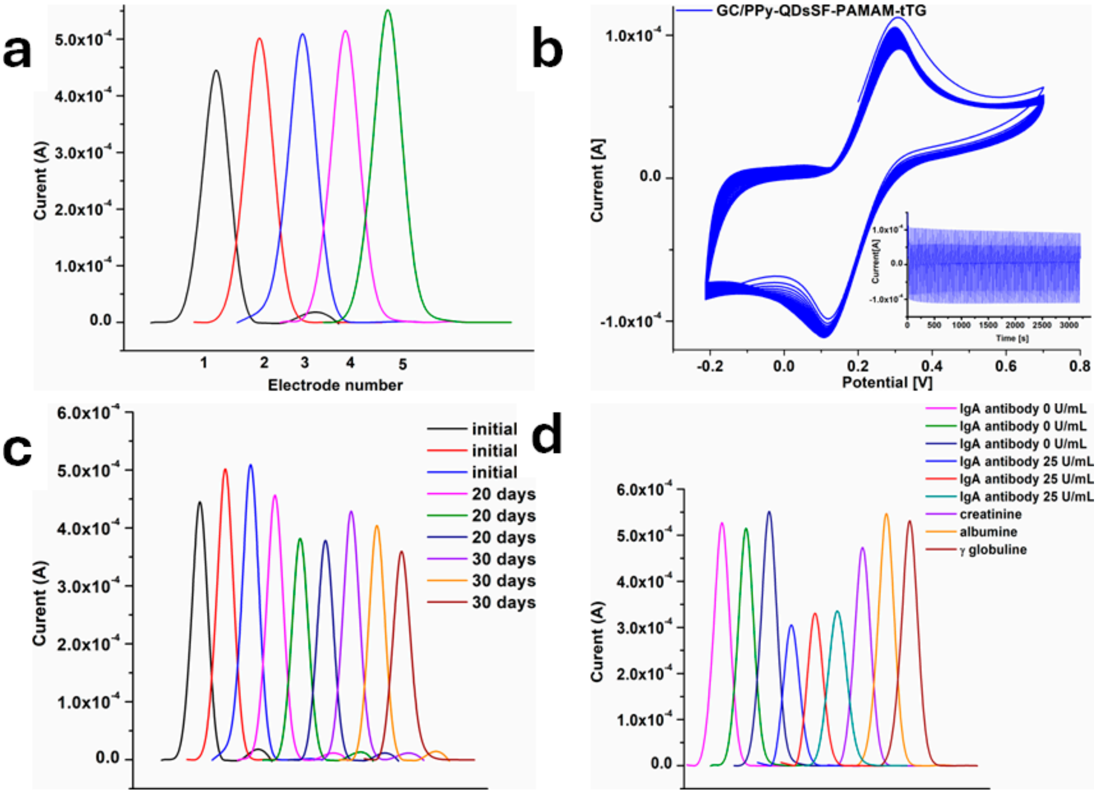

Figure S1 a) SWV curves for five GC/PPy-QDsSF-PAMAM-tTG electrodes scanned in PBS with Fe^{2+}/Fe^{3+} redox couple, b) CV of modified electrode, 100 consecutive cycles, c) SWV curves of modified electrode after different time of storage at 4°C, d) SWV curves after modified electrode incubation with anti-tissue antibody 0 U/mL, albumin, creatinine, and γ globulin
